# Influence of motivation on the perception of mathematics by secondary school students

**DOI:** 10.3389/fpsyg.2022.1111600

**Published:** 2023-01-19

**Authors:** Hassan Hossein-Mohand, Hossein Hossein-Mohand

**Affiliations:** ^1^Department of Pedagogy, Faculty of Teacher Training and Education, Universidad Autónoma de Madrid, Madrid, Spain; ^2^Department of Didactics of Mathematics, Faculty of Education and Sport, Universidad de Granada, Granada, Spain

**Keywords:** secondary education, academic performance, learning, motivation, mathematics

## Abstract

Motivation, the teacher–student relationship, the use of resources, and the time spent studying, in addition to the family economic and social context, are some of the factors that affect academic performance and directly influence student failure. This paper evaluates the motivation in mathematics students’ performances by analyzing indicators of the mathematics learning dimensions. A total of 2,018 secondary students were evaluated in this cross-sectional study. Motivation, teaching, resources, and study time were analyzed with a validated 20-item questionnaire. Statistical analysis revealed that student motivation appears to be significantly related to perceptions of teaching practices and the use of resources for study. Students with high motivation have positive perceptions of teaching practices. Gender differences were not observed. In addition, the motivation indicator allowed for grouping students into various motivational profiles.

## 1. Introduction

In general, secondary school students do not develop mathematical competence correctly due to their difficulties with understanding the basic theoretical concepts and the proper interpretations of data (Cipkova et al., 2019). Moreover, a negative perception of the subject, which is dependent on the interests of teenagers (Husein et al., 2018; Abin et al., 2020), has a negative impact on both a student’s academic performance (Ozhan and Kocadere, 2020) and their choice of STEAM studies (Hine, 2018).

### 1.1. Academic performance and student failure

Academic achievement is primarily associated with educational attainment (Williams et al., 2022). Therefore, grades are one of the reliable reference indicators of a student’s academic performance (Neuendorf et al., 2022). Moreover, a student regularly finishing their math homework was a significant predictor of academic performance (Xu, 2022). However, the amount of homework completed in secondary school did not have an association with a student’s academic performance (Tsang et al., 2022).

On the other hand, variables responsible for low academic performance can be classified into internal and external variables. Internal variables are associated with cognitive factors such as aptitude, intelligence, cognitive style, and personality factors such as motivation (Lim, 2021; Mercader Rubio et al., 2022), self-esteem (Smith et al., 2022), and self-confidence (Estrada-Madariaga, 2017; Vinni-Laakso et al., 2019). Several findings have suggested positive relationships between academic performance and emotional intelligence (Buenrostro and Radinsky, 2019).

External variables include socioeconomic status (Bardach et al., 2022; Musaddiq et al., 2022; Williams et al., 2022), family (Stavrulaki et al., 2021), and economic and school context (Siebecke and Jarl, 2022; Tan, 2022). With respect to social context, marginality, poverty, delinquency, drug use, etc., stand out (Perdereau-Noel et al., 2017; Martin et al., 2022). As for school context, a high ratio in a student’s classroom (INEE, 2021) and the role of teachers (Kim, 2020) stand out. In 2021, Spanish State School Board stated that low academic performance generally led to student failure.

One of the consequences of low academic performance and student failure is early school dropout (Cuenca Carrión et al., 2021). In 2019 and 2021, Spanish State School Board reports defined early school dropout as “the percentage of young people between 18 and 24 years of age whose highest academic qualification is Compulsory Secondary Education and who do not continue their education.” These data were provided by Eurostat from a labor force survey. For 2020, Europe set an early dropout rate of below 10% (15% for Spain) as a target (Cuenca Carrión et al., 2021).

### 1.2. Influences of academic factors

Numerous studies have analyzed the effects of educational environment on a student’s academic performance. Warwick et al. (2019) highlighted the teacher–student relationship as the most relevant element of performance (Warwick et al., 2019), and the scientific literature shows that the influences of personal motivation and classroom climate are key elements (Ozhan and Kocadere, 2020; Prieto, 2020; Rojo-Robas et al., 2020).

Results from other studies appear to confirm that the use of technology for educational purposes for learning mathematics is not significant in the academic performance of secondary mathematics students (Hossein-Mohand et al., 2021a). However, other studies have suggested that the excessive use of technologies has an influence on student failure in high school (Gómez-García et al., 2020a).

#### 1.2.1. Influence of the teacher–student relationship

The role of a teacher is vital in the teaching–learning process. A motivated teacher introduces technological (Gómez-García et al., 2020b), pedagogical, and methodological innovations in the teaching of mathematics (Trujillo-Torres et al., 2020a). Their role is fundamental to adequately assessing the classroom climate and to developing interventions that favor safe learning environments (Quijada et al., 2020). However, some authors have suggested a significant association between truancy and a negative classroom climate (Rathmann et al., 2018). In this sense, inadequate intervention is associated with an increase in disruptive behaviors (Lerang et al., 2019).

Regarding groupings of students within a classroom, several authors have argued that natural distributions are produced by antagonistic acceptance–rejection and popularity–unpopularity criteria (Reihenova, 2018; Laninga-Wijnen et al., 2019; Tereshchenko et al., 2019). Scientific evidence supports that the popularity–unpopularity criteria clustered similar pairs of both high and low performers and the effects of like-peer groupings that significantly impact students in ethnically mixed classrooms (Keller and Takacs, 2019). Additionally, boys with both high and low academic performance levels have significantly higher risks of being bullied than girls with the same academic profiles (Bergold et al., 2020).

However, the literature has established significant associations between the teacher–student relationship and student academic achievement (Holzberger et al., 2019; Klapproth and Fischer, 2019). Moreover, good teacher–student communication allows teachers to adequately assess their students’ mathematical competencies (Sánchez-Matamoros et al., 2019; Uner and Akkus, 2019) and develop effective pedagogical strategies within the classroom (Van-Kleij, 2019; Hossein-Mohand et al., 2021b).

The teacher–student relationship is significantly positively associated with the methodology used by the teacher. However, Roorda et al. (2019) pointed out that if a student has a bad relationship with their teacher, they also have a negative perception of the corresponding subject. This may explain the results showing low performance levels in mathematics in secondary schools (Lazarides et al., 2019; Ozdemir and Ozdemir, 2019). Moreover, the teacher–student relationship has a major influence on socioeconomically disadvantaged groups of students (Munter and Haines, 2019).

However, discrepant positions regarding the impact of the teacher–student relationship have been observed. Timmermans and Rubie-Davies (2018) suggested that the overall effects for the majority are insignificant, but for a minority, they could be very significant. In contrast, Goellner et al. (2018) claimed that long-lasting relationships between a student and teacher over time (in primary and secondary education) broaden the spectrum of dyadic effects on the student body. An example of the effect of this relationship is its impact on both academic and non-academic motivation in students (Trujillo-Torres et al., 2020b).

#### 1.2.2. Influence of motivation

In addition to the inherent capacity of a student, academic performance in mathematics has significant associations with self-discipline and perseverance (Hagger and Hamilton, 2019). The resilience needed to cope with learning this subject is supported by motivation (Sagone et al., 2020). Therefore, in this multifactorial analysis, it is worth highlighting the role of motivation as a fundamental variable for learning mathematics (Rojo-Robas et al., 2020; Trujillo-Torres et al., 2020b).

Intrinsic motivation arises from an individual’s own motivation by providing enjoyment in the face of challenges (Diseth et al., 2020). This motivation increases with personal development and perseverance and strengthens an individual’s resilience (Cheng, 2019). External motivation, on the other hand, stimulates an individual through different types of external rewards and recognition (Liu et al., 2020a). These stimuli detract from the intrinsic value of the activity performed and negatively affect a student’s performance (Gillison et al., 2019).

Different findings have suggested that extrinsic motivation has a significant negative impact on intrinsic motivation (Cheng, 2019). However, other authors have claimed that both types of motivation affect each other (Hidi et al., 2019; Liu et al., 2020a,b) and that this effect is not summative but multiplicative. Furthermore, they have pointed out that the effect of extrinsic motivation depends on an individual’s level of intrinsic motivation (Sheldon and Prentice, 2019). Past scientific findings have shown that the effect of a reward is negative only if the level of intrinsic motivation of a learner is high (Liu et al., 2020b). Otherwise (i.e., for unmotivated individuals or for individuals with very low levels of intrinsic motivation), rewards or instrumental motivation are important stimuli that arouse interest in a task’s “situational interest,” reinforce intrinsic motivation, and support an individual’s academic performance (Liu et al., 2020a).

Similarly, several authors have suggested significant correlations between autonomous motivation and positive perceptions toward studies in STEAM (Hagger and Hamilton, 2018; Hodis, 2018). They have pointed out significant positive correlations between students’ autonomous and controlled motivations and between students’ socio-cognitive beliefs toward scientific activities and their academic performance in these areas. On the other hand, there is evidence that shows that external stimuli (rewards, prizes, etc.) can impact superficial, non-durable cognitive effects. On the other hand, internal stimuli (commitment, self-improvement, etc.) can provoke deep, permanent cognitive effects and motivate students in a decisive way (Rican et al., 2022).

In terms of gender, the difference in motivational perceptions regarding mathematics has impacted the low number of young women studying STEAM (Hsieh, 2019; Vinni-Laakso et al., 2019). Possible causes of this include gender roles (Ehrtmann and Wolter, 2018; Steegh et al., 2019), which are influenced by sociocultural factors (Melak and Singh, 2021). The 2020 report by the Spanish Youth Institute (INJUVE) concluded that gender and sexual orientation are associated with the choice of STEAM in university studies (INJUVE, 2020). This report argued that, in general, the female gender and the homosexual collective show preferences toward artistic and humanistic studies, while the male gender prefers STEAM studies.

However, interest in STEAM among female students is positively correlated with the resources employed, time devoted to studying, and career prospects (Loh et al., 2019). In this sense, mathematics teachers can motivate students with appropriate pedagogical and methodological interventions (Vergara et al., 2019). Effective interventions significantly impact the mathematical competence acquired by a student (Garrido-Yserte et al., 2019; Levrini et al., 2019) and can favor their interest in STEAM (Barth et al., 2018). Preventing and acting against demotivation and avoiding consequent early school dropout is an arduous task that requires specific training for mathematics teachers (Gil et al., 2019; Hossein-Mohand et al., 2021b).

#### 1.2.3. Justification

The early school dropout rate for Spain (20.2%) is well above the European average (11.8%). The Autonomous City of Melilla (a Spanish city in north of Africa with a special administrative structure) has reached an extreme value in this respect, with an early school dropout rate of 22.8% (Cuenca Carrión et al., 2021). Since 2010, the A.C. of Melilla is among the two European cities with the highest rates. Although there was evidence of a decrease in these rates for the period 2008–2018, they were still far from attaining the European Council’s target of 10% by 2020 (Martínez-Abad et al., 2020). On the other hand, the gross secondary school graduation rate in Spain is 78.8%, but in the A.C. of Melilla, with the second lowest rate in the country, it is 52.7% (67.5% for females and 48.1% for males). Furthermore, the PISA 2018 report indicated that 75% of Spanish students had the minimum level of competence in mathematics, compared to the European average of 78% (Gamazo and Martínez-Abad, 2020). In terms of ranking in autonomous regions, the two lowest values were found in the autonomous cities of Ceuta (44%) and Melilla (53%) (INEE, 2021).

In this context, a cross-sectional analysis of academic variables that could influence the motivational profile of secondary and high school mathematics students is proposed. Due to the general profile of a student body, only the possible effect of extrinsic or instrumental motivation is evaluated. This quantitative study also evaluates the teacher’s role and the resources and study time of students.

The main objective of the present study was to determine the effect of motivation on the mathematics students in the A.C. of Melilla through the analysis of indicators of the dimension “Learning Mathematics.” The associated specific objectives are: first, to examine the relationship between the variables related to gender, educational level, teaching, study time, resources employed, and extrinsic motivation, and second, to establish the optimal number of clusters necessary to subdivide the sample according to the motivational profiles of the mathematics students in the A.C. of Melilla.

The hypotheses associated with personal and academic factors are as follows:

H1. The variables associated with the indicators—instrumental or extrinsic motivation, teaching, study time, employment of resources for study—and the variables—educational level and gender—do not have the same influence on the same secondary students, nor do they influence these students in the same way.

H2. There will not be a statistically significant correlation between each pair of the variables (gender and educational level) and the indicators (teaching, study time, resources used, and extrinsic motivation).

H3. The regressors (teaching, study time, employment of resources for study, educational level, and gender) do not significantly influence instrumental or extrinsic motivation through either main or interaction effects.

H4. There are no statistically significant differences between the genders of the students and their educational level, teaching, study time, resources used, and motivation.

## 2. Materials and methods

The present cross-sectional study is a non-experimental, *ex post* facto method which uses a closed questionnaire as the data collection instrument. Mathematics grades are used as a reference predictor for students’ mathematics academic performance (Barca et al., 2011; Córdoba et al., 2012).

### 2.1. Participants

The study sample corresponds to mathematics students in the A.C. of Melilla. The study population was obtained by applying the following inclusion criteria: (1) being under 18 years old, (2) residing in the City, and (3) studying in secondary and high school during the 2018/2019 academic year. With these criteria, the population amounts to 5,875 individuals (50.84% of which are female). The sample selection criterion was comprised of a non-probabilistic convenience sampling.

The initial sample had 2,039 students, but an initial analysis identified 21 incomplete questionnaires, and these were removed from the present study. Finally, a total of 2,018 students (53.40% females) from all schools and educational levels in the C. A. of Melilla participated in this study. The sample was differentiated by educational levels, compulsory secondary schools, and post-16 education, as follows: 1 secondary school (417), 2 secondary school (473), 3 secondary school (394), 4 secondary school (417), 1 Baccalaureate (233), and 2 Baccalaureate (84).

### 2.2. Instrument

The present study is part of a broad investigation focused on secondary and high school students in the A.C. of Melilla. An instrument with 135 closed-ended items of six dimensions and 31 indicators was used in this study, and it followed the Rosenbluth et al. (2016) procedure for the development of the questionnaire.

This work shows partial results related to 20 items of the dimension “B. Learning Mathematics” of the general questionnaire. It was taken to determine the possible relationship between the figures for the mathematics teacher, academic motivation, study time, and the use of different resources to study. The variables analyzed, their relationship with the corresponding indicators, and the dimensions are shown in Table 1.

**TABLE 1:** Relation between motivation study dimensions, indicators, and items.

Dimension	Indicators	Code	Items
A. General data	A.1 Student’s data	ECC	Are you a boy or a girl?
		NEC	What level of education are you studying?
B. Aprendizaje de las matemáticas	B.4 Teaching PMT= PMC+PME+PMR+ +PMM+PCT+PRE	PMC	Does your math teacher create an appropriate climate for learning?
		PME	Does your math teacher explain well in class?
		PMR	Does your math teacher review what was explained the day before?
		PMM	Does your math teacher send homework?
		PCT	Does your math teacher correct the homework?
		PRE	Does your math teacher do a review before the exam?
	B.5 Study time ST=LJM+VSM	LJM	From monday to thursday, how many hours do you spend each day studying mathematics?
		VSM	On weekends, how many hours do you spend each day studying mathematics?
	B.6 Resources ER=ULT+UAE+ +UVI +UAI	ULT	Do you use the textbook to study mathematics?
		UAE	Do you use your notebook or class notes to study mathematics?
		UVI	Do you use internet video tutorials to study mathematics?
		UAI	Do you use notes taken from the internet to study mathematics?
	B.8 Motivation MO=MRP+MGA+MEF+ +MFM+MAM+MPM	MRP	When studying mathematics, are you motivated by your family’s gifts?
		MGA	Are you motivated to study mathematics because you enjoy the subject?
		MEF	Are you motivated to study mathematics to succeed in the future?
		MFM	Does your family motivate you to study mathematics?
		MAM	Do your friends motivate you to study mathematics?
		MPM	Does your teacher motivate you to study mathematics?

Likert-scale for study variables: gender (ECC) (1, Female; 2, Male); educational level (NEC) (1, 1 Secondary; 2, 2 Secondary; 3, 3 Secondary; 4, 4 Secondary; 5, 1 High School; and 6, 2 High School); working days (LJM) and public holidays (VSM) (1, none; 2, less than 1 h; 3, from 1 to 2 h; and 4, more than 2 h); For all other items, (1, none; 2, a little; 3, enough; and 4, a lot).

#### 2.2.1. Instrument validation

The questionnaire was previously subjected to content validation by the judgment of ten experts with more than 15 years of academic experience (four researchers in mathematics education, three secondary school principals, and four heads of mathematics departments) in terms of the level of writing, as well as the degree of appropriateness of the items used. In addition, a pilot test was administered to 20 students to detect the last aspects that were susceptible to improvement.

Finally, the internal data matrix was validated by verifying that, in its composition, the conjunction of the heterogeneous items was coherent, using the Kaiser–Guttman criterion and the Tucker–Lewis index. First, the optimal number of dimensions of the instrument was assessed through the Kaiser–Guttman criterion and used to identify the optimal number of axes. The initial number was structured around 6 dimensions. However, the algorithm established that the optimal number of axes is three.

The generalized low-rank models (GLRM) and principal component analysis (PCA) were used for validation, taking the following parameters as references:

• loss = “Quadratic.”
• regularization_x = “L1.”
• gamma_x = 0.5.
• gamma_y = 0.
• max_iterations = 1,000.

The results obtained were as follows:

• The instrument can be reduced to four dimensions.
• The first dimension explains 24% of the variance, the second 18%, the third 16%, and the fourth 15%.

Based on the previous results, it was confirmed that, in order to optimize the instrument, it could be reduced from 6 to 4 dimensions.

To complete the validation, a double exploratory factor analysis was carried out, with the first one testing the previous statement about the dimensions and the second, using varimax rotation, identifying the variables that could be eliminated.

The results of the first exploratory factor analysis are as follows:

• Mean item complexity = 1.2.
• Test of the hypothesis that five factors are sufficient.
• The degrees of freedom for the null model are 15 and the objective function is 0.15, with chi-square value of 302.63.
• The degrees of freedom for the model are −5 and the objective function is 0.
• The root mean square of the residuals (RMSR) is 0.
• The Tucker–Lewis index of factoring reliability = 1.052.
• Fit based upon the off-diagonal values = 1.

Considering the original dimensions of the instrument, and given that the Tucker–Lewis index of factoring reliability is higher than 0.9, the instrument can be optimized with the choice of five axes.

These axes were then compared with the six axes initially established in the original instrument. The Bayesian information criterion (BIC) was lower by taking five dimensions, which confirmed this as the optimal solution.

For the second exploratory factor analysis using varimax rotation, uniqueness was calculated, and the residual matrix was analyzed. The proportion of variability is denoted as communality. One way to calculate it is to subtract uniqueness from one. An appropriate factor model results in low values for uniqueness and high values for communality. These results were met for the validation of the instrument. As a result, seven variables were removed from the set.

For the calculation of the residual matrix, the following scripts were used:

• Lambda < data$loadings.
• Psx < diag(data.fa$uniquenesses).
• S < data.fa$correlation.
• Sigma < Lambda%*% t(Lambda) + Psx.

The results showed for all cases where values were close to 0, those variables were well-represented.

Finally, the power of the statistical test was calculated. For this purpose, the following procedure was used: Two datasets were defined, with x1 selecting only the variable gender and x2, teaching (with level = 0), and an analogous one, y, which selected the same type of variables, but took a PMT level of one. Then, Cohen’s d was used for both datasets (d estimate = 0.11). For calculation of the power calculations for the *t*-tests of the means, the d value was replaced by the d estimate above and a power of 95% was defined. The results showed that 1,955 subjects (*N* = 1,955,106) are necessary to detect significant differences in the study, a result that is otherwise within the sample size.

#### 2.2.2. Data set key variables

The analyses of the present work are conducted using the R language in R studio (R Studio_Team, 2020; R Core_Team, 2021) to evaluate in a gender-differentiated analysis the possible association between the extrinsic motivation (MO) indicator and the academic variables that are detailed below.

The initial dataset consisted of 2018 observations and 137 variables. However, for this study, only the following variables and indicators are taken into consideration (Table 1):

The MO indicator groups together six questions from the global instrument belonging to the dimension “B. Learning Mathematics” (items B.82–B.87). The responses are adjusted based on a Likert scale of four (1, none; 2, a little; 3, enough; and 4, a lot).

The teaching indicator (PMT) focuses on a student’s perception of their mathematics teacher’s practices and includes six questions from the global instrument (items B.41–B.46). The study time indicator (ST) is associated with the average time spent by a student studying mathematics on working days (LJM) and on public holidays (VSM) (items B.52 and B.53). A different Likert scale of four is used for these items (1, none; 2, less than 1 h; 3, from 1 to 2 h; and 4, more than 2 h). As for the resources (RE) indicator, it focuses on the different resources (technological and non-technological) used by a student for learning mathematics and includes four questions from the global instrument (items B61–B.64) as references. The responses are adjusted based on a Likert scale of four (1, none; 2, a little; 3, enough; and 4, a lot).

This study also aims to assess whether there are gender differences (ECC) and the influence of educational level (NEC). The Likert-scale for the study variables is: ECC (1, female and 2, male); NEC (1, 1 secondary; 2, 2 secondary; 3, 3 secondary; 4, 4 secondary; 5, 1 high school; and 6, 2 high school).

Table 1 provides an overview of the interconnectedness of the above elements.

##### 2.2.2.1. Data collect process

The data collection procedure was complex as it was conducted during school hours, but it was the only one that guaranteed the reliability of the data. This required the active collaboration of the management, the ICT coordinator, and the mathematics and technology departments of each center to implement the questionnaires with the least possible impact on school operations. It was also necessary to organize classroom and technological resources. The reliability of data collection was guaranteed using Google Forms, and a procedure was chosen to eliminate coder transcription errors (Cohen and Manion, 1990). The questionnaires were circulated in a computer classroom during school time to avoid bias, and they were given to all the secondary and high schools in the A.C. of Melilla.

Throughout the data collection process, a mathematics teacher was always assigned to each school to answer questions about the questionnaire, and another teacher from the technology department was assigned to deal with technical issues. At the end of the online questionnaire, each student returned to their classroom to continue with their studies.

The variables analyzed in this study, their relationship with the corresponding indicators, and the dimensions are shown in Table 1.

### 2.3. Ethics statements

Because our participants are under 18 years-old, the questionnaire was reviewed by the local educational authorities and received authorization from the Provincial Director of the Ministry of Education to distribute the questionnaires in the educational centers of the city during school hours. In addition, all subjects who participated voluntarily were fully informed of the nature of the research.

The study complies with the ethical criteria of the Declaration of Helsinki. In addition, it follows the AERA (American Educational Research Association) code of ethics for research in education.

## 3. Results

In our previous studies, we evaluated the incidence of some variables on performance and the relationships between them. However, this study aims to globally analyze the effect of motivation on some factors. Therefore, it was considered appropriate to group the variables of the dimension “B. Learning Mathematics” around the corresponding indicator (see Table 1):

• Gender (ECC);
• Educational level (NEC);
• Teaching (PMT): PMC + PME + PMR + PMM + PCT + PRE;
• Study time (ST): LJM + VSM;
• Resources used (ER): ULT + UAE + UVI + UAI;
• Motivation (MO): MRP + MGA + MEF + MFM + MAM + MPM.

We note that the grouping of the variables of the motivation indicator quantifies their overall effect. The present study does not attempt to distinguish between the intrinsic and extrinsic motivation of the subjects. Furthermore, we note that the above groupings are not to be interpreted as a linear combination of variables.

Complementarily, each of the independent variables were factored and converted into numerical variables. They were then binned as follows:

• If the mean of PMT > 10.4∼1;
• If the mean of ER > 5.8∼1;
• If the mean of MO > 10∼1;
• Otherwise, 0. It is important to note that the values 0 and 1 were factored. This latter procedure was performed to test the Mann–Whitney U-test values.

**FIGURE 1:**
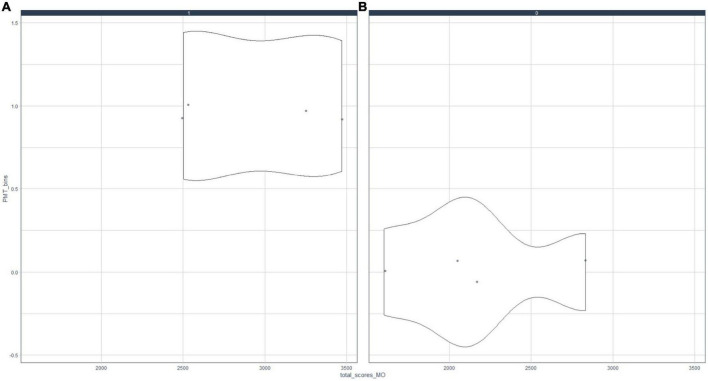
Relationship between motivation (MO) and teaching (PMT). Association of motivation and teaching indicators; **(A)** MO = 0, **(B)** MO = 1.

**FIGURE 2:**
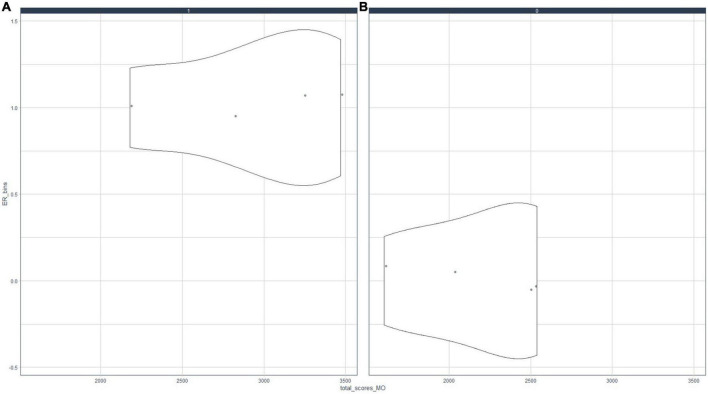
Relationship between motivation (MO) and resources used (ER). Association of motivation and resources for study; **(A)** MO = 0, **(B)** MO = 1.

To achieve objective ¨OE1.1 To examine MO, PMT, ST, ER, NEC, and ECC variables and their relationships¨; and ¨OE1.2 To determine the optimal number of clusters necessary to subdivide the sample around the motivational profiles of the mathematics students of the A.C. of Melilla¨, the relationships between indicators B4 and B8 were analyzed. Figures 1, 2 presented in-depth correlation analyses of the motivational profile.

Figure 1 shows that the behavior of motivated subjects with respect to PMT is significantly different from when they are not motivated, i.e., if a student is highly motivated (academically, by rewards, by their family, etc.), they will have a high perception of the teaching practices of their mathematics teacher. Otherwise, a student’s perceptions about teaching methodologies will be negative. Similarly, Figure 2 illustrates that the MO and RE scores are strongly positively correlated. In general, if students are motivated, they tend to use different resources to study. The impact is different for unmotivated students, who tend to obtain low values in using different resources for studying mathematics.

**FIGURE 3:**
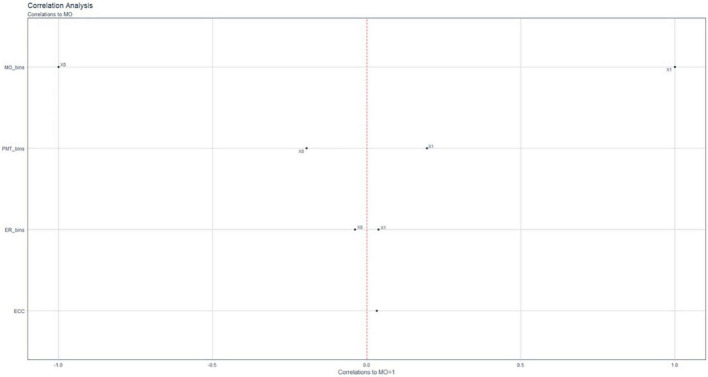
Correlation Funnel. Scores of studying and teaching resources to the motivation indicator.

Another instrument used to evaluate the motivational profile of mathematics students in the A.C. of Melilla is the funnel correlation analysis shown in Figure 3.

Figure 3 shows that the variables MO, PMT, and ER are binary (yes/no), i.e., the subject’s motivation could be either No = 0 or Yes = 1. In the case of MO = 1, there is a strong correlation between a PMT of one and an ER of one. On the other hand, when MO takes a value of zero, there is a correlation with an ER of zero and a PMT of zero. These results imply that if the students are motivated, their perceptions of the teaching function improve and they use the appropriate didactic resources to study.

In the analysis differentiated by gender, our results suggest that there are no gender differences in the motivation associated with learning mathematics. Therefore, hypothesis H1 is rejected. “The relation between the variable MO with PMT, ST, ER, NEC, and ECC does not follow a similar pattern.”

**FIGURE 4:**
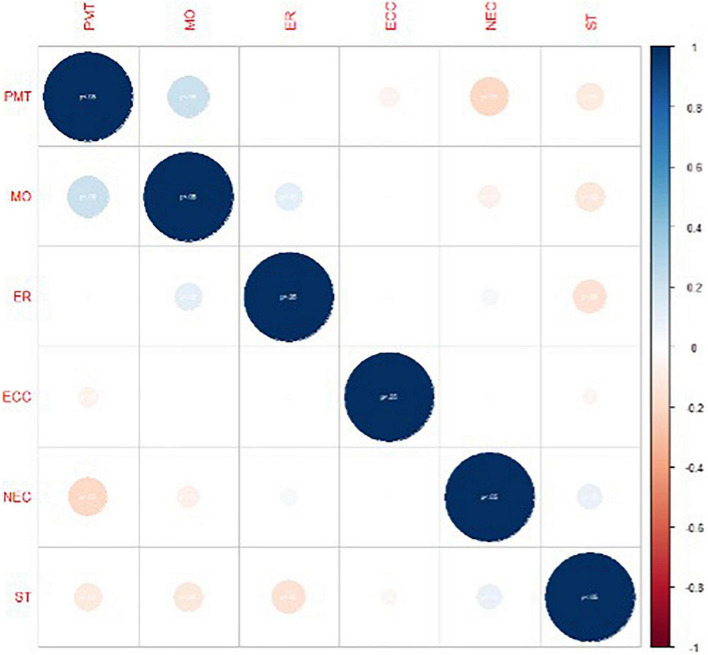
Correlation between the variables gender, educational level, teaching, study time, resources used, and motivation.

To investigate further, the correlations between the variables of gender, educational level, teaching, study time, different resources used, and motivation are evaluated. The results are shown in Figure 4.

Figure 4 shows that in most cases, the correlations are weak and inverse. In addition, the strongest correlation is between PMT and MO. Based on these results, the null hypothesis H2 is accepted.

In order to answer hypothesis H4 on the existence of significant differences between the genders of students in relation to educational level, teaching, study time, different resources used, and motivation, the Mann–Whitney U-test is used. The results show that there are only significant differences between males and females with respect to the teaching variable (W = 547,220, *p*-value = 0.02431). Consequently, the null hypothesis H4 is partially rejected.

**FIGURE 5:**
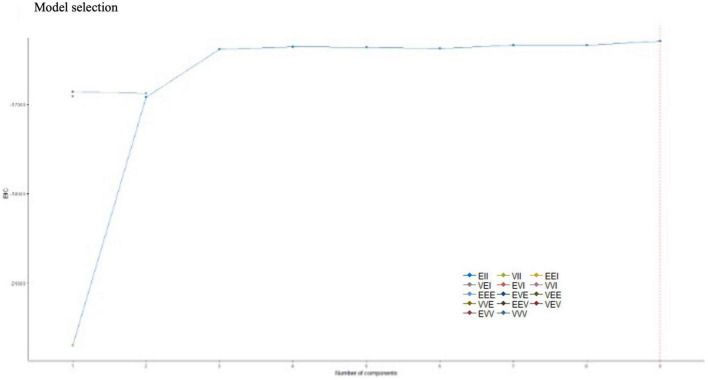
Optimal Cluster. Selection of the optimal number of clusters.

A study is then carried out to answer the research question: can mathematics students be grouped into clusters according to their motivational profile, and if so, what would be the optimal number of clusters? A principal component analysis (PCA) is performed to evaluate possible groupings, as shown in Figure 5.

Figure 5 illustrates that the optimal number of optimal clusters to subdivide the sample is nine. In other words, the mathematics students in the A.C. of Melilla can be grouped around nine differentiated profiles. Furthermore, the algorithm employs 14 classifier models for its analysis: EII, VII, EEI, VEI, EVI, VVI, EEE, EVE, VEE, VVE, EEV, VEV, EVV, and WWW, of which the optimal model is EII. Therefore, hypothesis H5 is rejected. “It is not possible to classify students according to their motivational profile.” This interesting finding has sparked the authors’ interest in conducting a new comprehensive and independent study to determine the nature and classification of students according to their motivational profiles, and it will be published soon.

To address objective 2, to determine if the variables PMT, ST, ER, and ECC have a significant impact on MO, a linear regression analysis is performed for the motivation variable. To quantify the weight of the most influential regressors, the analysis also identifies the level of each variable and their combined role. In other words, the effect of each variable as the main element and the interaction of two or more variables are analyzed. Table 2 shows the results.

**TABLE 2:** Linear regression model for the motivation (MO) variable.

Residuals	Min.	1Q	Median	3Q	Max.
	−9.2418	-1.8595	0.0788	1.9389	8.8876
Intercept		Estimate	Std. error	*T*-value	Pr(>|t|)
		0.72165	5.76703	0.125	0.9004
ECC		4.87291	6.70614	0.727	0.4675
NEC1		3.62759	7.42034	0.489	0.6250
NEC2		-9.20725	8.66266	-1.063	0.2880
NEC3		8.57115	7.06211	1.214	0.2250
NEC4		11.60347	6.67678	1.738	0.0824
NEC5		5.09159	6.41347	0.794	0.4274
PMT		0.46504	0.42184	1.102	0.2704
ST		1.26475	1.68392	0.751	0.4527
ER		1.91714	0.92687	2.068	0.0387*
ECC:NEC1		0.20359	9.02970	0.023	0.9820
ECC:NEC2		7.84071	12.6894	0.618	0.5367
ECC:NEC3		-7.99019	8.45670	-0.945	0.3449
ECC:NEC4		-10.94995	8.11424	-1.349	0.1773
ECC:NEC5		-2.84826	7.71548	-0.369	0.7120
ECC:PMT		-0.30222	0.51635	-0.585	0.5584
NEC1:PMT		-0.07534	0.57346	-0.131	0.8955
NEC2:PMT		1.69388	0.75686	2.238	0.0253*
NEC3:PMT		-0.34390	0.54462	-0.631	0.5278
NEC4:PMT		-0.38254	0.50958	-0.751	0.4529
NEC5:PMT		0.24417	0.50362	0.485	0.6279
ECC:ST		-1.39276	2.19086	-0.636	0.5250
NEC1:ST		-0.73305	2.17870	-0.336	0.7366
NEC2:ST		3.63684	2.70991	1.342	0.1797
NEC3:ST		-1.94971	1.96479	-0.992	0.3212
NEC4:ST		-1.75090	2.01618	-0.868	0.3853
NEC5:ST		-1.26409	1.84707	-0.684	0.4938
PMT:ST		-0.02932	0.13224	-0.222	0.8245
ECC:ER		-1.53430	1.06049	-1.447	0.1481
NEC1:ER		-1.71432	1.21535	-1.411	0.1585
NEC2:ER		1.01890	1.51842	0.671	0.5023
NEC3:ER		-2.20873	1.12209	-1.968	0.0492*
NEC4:ER		-2.16956	1.05219	-2.062	0.0393*
NEC5:ER		-1.86158	1.01242	-1.839	0.0661
PMT:ER		-0.10950	0.07006	-1.563	0.1183
ST:ER		-0.36808	0.26844	-1.371	0.1705
ECC:NEC1:PMT		-0.02569	0.70869	-0.036	0.9711
ECC:NEC2:PMT		-1.61442	1.13112	-1.427	0.1537
ECC:NEC3:PMT		0.52268	0.68982	0.758	0.4487
ECC:NEC4:PMT		0.48666	0.66263	0.734	0.4628
ECC:NEC5:PMT		-0.41399	0.63213	-0.655	0.5126
ECC:NEC1:ST		0.86789	2.77575	0.313	0.7546
ECC:NEC2:ST		-2.68802	3.72318	-0.722	0.4704
ECC:NEC3:ST		3.00035	2.57308	1.166	0.2437
ECC:NEC4:ST		1.93040	2.59628	0.744	0.4573
ECC:NEC5:ST		1.35530	2.41942	0.560	0.5754
ECC:PMT:ST		0.09677	0.17594	0.550	0.5824
NEC1:PMT:ST		-0.02337	0.17953	-0.130	0.8964
NEC2:PMT:ST		-0.64541	0.23273	-2.773	0.0056**
NEC3:PMT:ST		0.07599	0.15880	0.478	0.6323
NEC4:PMT:ST		-0.01969	0.16396	-0.120	0.9044
NEC5:PMT:ST		-0.06007	0.15656	-0.384	0.7012
ECC:NEC1:ER		1.06909	1.45494	0.735	0.4626
ECC:NEC2:ER		-0.14862	2.07678	-0.072	0.9430
ECC:NEC3:ER		2.45232	1.33431	1.838	0.0662
ECC:NEC4:ER		1.77181	1.26590	1.400	0.1618
ECC:NEC5:ER		1.47947	1.20187	1.231	0.2185
ECC:PMT:ER		0.11956	0.08449	1.415	0.1572
NEC1:PMT:ER		0.11668	0.09507	1.227	0.2198
NEC2:PMT:ER		-0.25277	0.13334	-1.896	0.0582
NEC3:PMT:ER		0.14079	0.08872	1.587	0.1127
NEC4:PMT:ER		0.10273	0.08257	1.244	0.2136
NEC5:PMT:ER		0.06887	0.08058	0.855	0.3929
ECC:ST:ER		0.39802	0.35828	1.111	0.2667
NEC1:ST:ER		0.30880	0.36652	0.843	0.3996
NEC2:ST:ER		-0.61635	0.51895	-1.188	0.2310
NEC3:ST:ER		0.52585	0.32000	1.643	0.1010
NEC4:ST:ER		0.34118	0.32687	1.044	0.2970
NEC5:ST:ER		0.43713	0.29126	1.501	0.1340
PMT:ST:ER		0.01812	0.02196	0.825	0.4090
ECC:NEC1:PMT:ST		-0.06101	0.22973	-0.266	0.7906
ECC:NEC2:PMT:ST		0.53104	0.33868	1.568	0.1170
ECC:NEC3:PMT:ST		-0.23530	0.21419	-1.099	0.2721
ECC:NEC4:PMT:ST		-0.07418	0.21714	-0.342	0.7327
ECC:NEC5:PMT:ST		0.01289	0.20803	0.062	0.9506
ECC:NEC1:PMT:ER		-0.09247	0.11574	-0.799	0.4244
ECC:NEC2:PMT:ER		0.17582	0.18449	0.953	0.3407
ECC:NEC3:PMT:ER		-0.19979	0.11127	-1.796	0.0727
ECC:NEC4:PMT:ER		-0.09544	0.10671	-0.894	0.3712
ECC:NEC5:PMT:ER		-0.04908	0.09971	-0.492	0.6226
ECC:NEC1:ST:ER		-0.31867	0.47341	-0.673	0.5009
ECC:NEC2:ST:ER		0.32636	0.67285	0.485	0.6277
ECC:NEC3:ST:ER		-0.71982	0.42640	-1.688	0.0915
ECC:NEC4:ST:ER		-0.27589	0.42827	-0.644	0.5195
ECC:NEC5:ST:ER		-0.43524	0.39115	-1.113	0.2660
ECC:PMT:ST:ER		-0.03216	0.02979	-1.080	0.2804
NEC1:PMT:ST:ER		-0.01292	0.03048	-0.424	0.6717
NEC2:PMT:ST:ER		0.11112	0.04439	2.503	0.0124*
NEC3:PMT:ST:ER		-0.03333	0.02673	-1.247	0.2126
NEC4:PMT:ST:ER		-0.00677	0.02754	-0.246	0.8058
NEC5:PMT:ST:ER		-0.01477	0.02533	-0.583	0.5599
ECC:NEC1:PMT:ST:ER		0.02507	0.03961	0.633	0.5269
ECC:NEC2:PMT:ST:ER		-0.07741	0.05973	-1.296	0.1952
ECC:NEC3:PMT:ST:ER		0.06329	0.03638	1.739	0.0821
ECC:NEC4:PMT:ST:ER		0.01589	0.03687	0.431	0.6666
ECC:NEC5:PMT:ST:ER		0.02189	0.03418	0.640	0.5220

Signif. codes: 0 “***”; 0.001 “**”; 0.01 “*”; 0.05 “.”; 0.1 “ “1; residual standard error: 2.807 on 1,943 degrees of freedom (DF); multiple R-squared: 0.1288.

Adjusted R-squared: 0.08618.

F-statistic: 3.023 on 95 and 1,943 degrees of freedom, *p*-value < 2.2 × 10–16.

Table 2 shows that the model is significant (*p*-value of <2.2 e–16), but it presents a low R2 (8.6% of the variation in student motivation depends on the variables ECC, NEC, PMT, ST, and ER). Less than 10% of the variability in motivation is explained by gender, educational level, teaching, study time, and use of different resources for study. We expect that there must be other factors associated with motivation that are not contemplated in the present paper and whose weight accounts for more than 90% of the variability.

The variables ER, NEC2: PMT, NEC3:ER, NEC4:ER, and NEC2: PMT:ST, NEC2:PMT:ST:ER had a significant contribution with respect to student motivation. In other words, the profiles that correlated highly significantly with motivation were as follows: (1) the student body, in general, employs different resources for study, and the highest correlations were found among students in 2 secondary school and 3 secondary school; (2) students in the 2 years of high school, in general; and (3) the highest scores were found among those who value teaching, dedicate time to study, and use different resources to learn mathematics.

Based on the above results, the null hypothesis H3 is partially rejected. “The regressors PMT, ST, ER, NEC, and ECC do not significantly influence MO, either through main or interaction effects.”

The low impact of the regressors in our study on motivation (<10%) suggests the existence of others of greater relevance that are affecting mathematics students in the A.C. of Melilla.

## 4. Discussion

This study aims to determine the possible relationship between the figures of the mathematics teacher, academic motivation, study time, and use of different resources to study.

The present paper shows a significant association between a student’s extrinsic motivation and their positive perception of their mathematics teacher (Figure 1). Similarly, this study suggests a strongly positively correlation between student motivation with the use of different resources to study (Figure 2). In this sense Liu et al. (2020b) evidenced that instrumental motivation reinforces intrinsic motivation and arouses “situational interest,” and it has an especially important impact on students with poor academic performance (Liu et al., 2020a). Other authors have suggested that an individual’s intrinsic and extrinsic motivation influence each other (Hidi et al., 2019; Liu et al., 2020b), producing a multiplicative effect on a student’s academic performance (Sheldon and Prentice, 2019). Roorda et al. (2019) pointed out that a good student–teacher relationship has a significant positive effect on a student’s perception of the subject. Additionally, this influence is highly significant in socioeconomically disadvantaged groups of students (Munter and Haines, 2019; Martin et al., 2022). Additionally, Warwick et al. (2019) suggested a relevant association between teacher–student relationship and academic failure. In this sense, if a student’s perception of their mathematics teacher is negative, their interest in the subject declines (Rojo-Robas et al., 2020). Consequently, their low academic results lead to school failure (Al-Shannaq and Leppavirta, 2020) and early school dropout (Cuenca Carrión et al., 2021). Other findings have pointed to associations between early school dropout with a negative classroom climate (Rathmann et al., 2018) and an increase in disruptive behaviors among high school students (Lerang et al., 2019).

In the analysis differentiated by gender to find possible correlations between gender, educational level, teaching, study time, different resources used, and motivation (Figure 3), our results suggest that there are no gender differences in motivation associated with learning mathematics. Other analysis shows that in most cases, the correlations are weak and inverse (Figure 4). Furthermore, the Mann–Whitney U-test shows that there are only significant differences between males and females with respect to their perceptions of their mathematics teachers.

In comparison to our results, the scientific literature shows contradictory findings. They point out the differences in motivational perceptions regarding mathematics in young women’s choices of undergraduate studies in STEAM (Hsieh, 2019; Vinni-Laakso et al., 2019). The low participation is due to the gender roles assumed by women during adolescence (Ehrtmann and Wolter, 2018; Steegh et al., 2019), which are influenced by sociocultural factors (Melak and Singh, 2021). However, the literature demonstrates that interest in STEAM among female students is positively correlated with different resources employed, time spent studying, and career prospects (Loh et al., 2019).

To further assess the possible relationship between the variables of study, in a linear regression analysis, the profiles that correlated highly significantly with motivation are the following: (1) the student body, in general, employs different resources for study, and the highest correlations were found among students in 2° secondary school and 3° secondary school; (2) students in the 2 years of high school, in general; and (3) the highest scores were found among those who value teaching, dedicate time to study, and use different resources to learn mathematics. The low impact of the regressors in our study on motivation (<10%) suggests the existence of others of greater relevance that are affecting mathematics students in the A.C. of Melilla. Along the same lines, other findings have shown a significant effect of technologies on academic performance (Gómez-García et al., 2020a). In addition, socioeconomic status (Bardach et al., 2022; Musaddiq et al., 2022; Williams et al., 2022) and family (Stavrulaki et al., 2021) are factors that should be contemplated.

The present manuscript quantifies the optimal clusters of mathematics students, which are grouped according to their motivational profile (Figure 5). This result is very interesting as it suggests that the mathematics teacher should attend to nine training realities in the classroom from the motivational point of view. In line with the above, Mora et al. (2020) recommended using appropriate teaching strategies that awaken the interest and participation of students in STEAM. In addition, the differences in preferences associated with gender should be contemplated (INJUVE, 2020). These actions could reduce the high early school dropout rate in the A.C. of Melilla (Roca Cobo et al., 2019).

## 5. Conclusion

The association between the study variables and motivation has followed a similar pattern. Therefore, the results have confirmed the possibility of classifying students into nine groups according to their motivational profile. On the one hand, the regressors of teaching, study time, employment of resources for study, and educational level have shown an influence on motivation, confirming our study hypothesis H3.

Our results have also shown a significant correlation between PMT and MO, accepting the null hypothesis (H2) there is no statistically significant correlation between each pair of variables (gender and educational level and teaching indicators, study time, resources used and motivation).

Finally, our results present certain limitations associated primarily with the cross-sectional nature of this study. Furthermore, the present study focuses only on mathematics in a specific geographical area.

## Data availability statement

The raw data supporting the conclusions of this article will be made available by the authors, without undue reservation.

## Ethics statement

The studies involving human participants were reviewed and approved by Dirección Provincial de Educación de Melilla. Written informed consent for participation was not required from the participants or their legal guardians/next of kin. Because the students were under 18 years-old, the questionnaire was previously reviewed and authorized by the local authorities of the Ministry of Education.

## Author contributions

HoH-M performed the formal analysis and conducted the validation. HaH-M wrote the first draft of the manuscript. Both authors contributed to conception of the study, manuscript revision, read, and approved the submitted version.
